# Efficacy of 17α- hydroxy progestrone on decreasing preterm labor in ART pregnancies: A randomized clinical trial

**Published:** 2013-10

**Authors:** Abbas Aflatoonian, Hoora Amouzegar, Razieh Dehghani Firouzabadi

**Affiliations:** *Department of Obstetrics and Gynecology, Research and Clinical Center for Infertility, Shahid Sadoughi University of Medical Sciences, Yazd, Iran.*

**Keywords:** *Preterm labor*, *Assisted reproductive technology*

## Abstract

**Background:** Preterm labor (PTL) is one of the most important causes in neonatal mortality and morbidity. Late preterm labor (34-36w) includes 75% of such birth. Assisted reproductive technology (ART) pregnant women are at increased risk of PTL.

**Objective: **The study has been undertaken to determine whether beginning and continuing 17-α hydroxy progesterone caproate can reduce risk of PTL or change neonatal mortality.

**Materials and Methods:** In a double-blind clinical randomized control trial, 106 women were treated by ART technique for their infertility and in gestational age at 16 weeks entered in our study. In one group, 17-α hydroxy progesterone caproate (Femolife) was injected intramuscularly every week until 36 weeks of gestation and in another group; placebo was injected from 16 until 36 weeks of gestetion. Data collected from pregnancy outcomes, infancy, and subsidiary problems were statistically analyzed by a questionnaire.

**Results: **The risk of PTL in placebo group was 2.48 higher than control group that was not significant (Cl: 0.81-9.94). Femolife side effect in case group was gestational diabetes and local complication was not frequent. NICU admission was not significantly different between groups.

**Conclusion:** Although it seems that 17-α hydroxy progesterone caproate does not cause significantly decrease in PTL in singleton ART gestations but any reduction of PTL in such high risk pregnancies may improve final gestational outcome. There is critical need for larger clinical trials to better understanding causes of PTL, specifically late preterm labor, to prevent mortality and morbidity in ART gestation.

This article extracted from Residential thesis. (Hoora Amouzegar)

Registration ID in IRCT: IRCT2012101611132N1

## Introduction

Preterm birth is declared by the World Health Organization (WHO) as a birth before 37 weeks of gestation, and its management and prevention remained as one of the problems in health policies. It was estimated that approximately 13 million preterm births occur around the world annually (1, 2). The incidence of preterm labor varies from 5-11% (3, 4). Many factors have been introduced for this complication of pregnancy such as maternal age and use of reproductive techniques which the second one is because of multiple pregnancy and singleton ones in comparison with spontaneous pregnancies are at double risk of preterm labor that it may be related to hormonal stimulation and mechanical procedures and gamete manipulation; culture environment and drugs in assisted reproductive technology (ART) (5-7). Other risk factors may be increasing maternal body mass index, smoking during pregnancy, and infections (8, 9).

Late preterm birth is defined as those gestational weeks composed approximately 70% of preterm birth (34-36). These infants predominantly suffer not only the immediate complications of prematurity but also long term sequelae such as neurodevelopmental disability (10). Besides increasing mortality and morbidity rates, the costs related to preterm birth and neonatal intensive cares are considerable. In United States in 1990, the costs of intensive care for preterm neonate estimated 10000 US$ weekly (11). It has been suggested that immunosuppressive was effected of progesterone with reproductive tract are at least partialy responsible for maintenance of semiallogenic implanting fetus. Progesterone mediated changes in T-cell gene expression have been associated with development of T-helper 2 (TH2) responses and Luckemia inhibitory factor (LIF) expression because a shift in intrauterine immune environment from TH2-TH1 has been linked with early spontaneous pregnancy loss.

The elevated intrauterine concentration of progesterone in early pregnancy may promote favorite pregnancy maintenance (12). The mechanism of the preterm labor may be due to complex interaction of many different hormonal effects (13). Progesterone plays an essential role in pregnancy progression (14). Progesterone has been used as a therapeutic agent for preterm labor since 1960 (15). While the exact mechanism of both term and preterm labor remains unclear, using progesterone is a therapeutic modality which plays an important role in the progression of pregnancy because of uterine quiescence (16).

This study was designed to investigate the effects of 17-hydroxy progesterone caproate on decreasing preterm labor in women who pregnant with ART technique.

## Materials and methods

In a double-blind clinical randomized control trial, which was done from October 2010 to October 2011. 106 women were investigated at Madar Hospital and Yazd Research and Clinical Center for Infertility. All women were treated by ART program for their infertility (with any different etiologies: male or female). The patients were entered in our study at 16 weeks of gestational age. All of them used progesterone in luteal phase and did not have twin or multiple pregnancies. They had neither history of preterm labor nor history of previous abortion. Women with history of chronic diseases (Diabetes mellitus, Hypertention, and Hypo or Hyperthyroidism) and uterine anatomical problems were excluded from this study.

**Figure 1:**
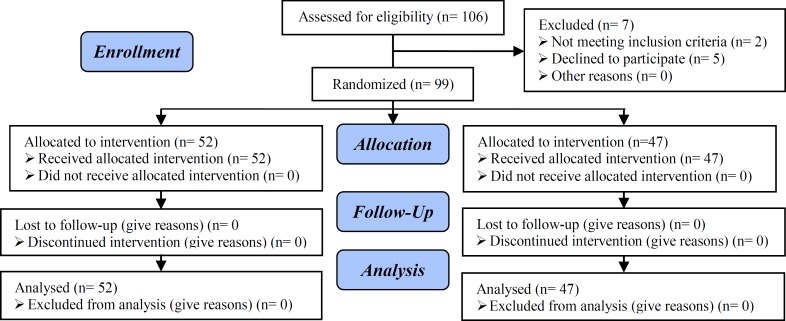
Flow char consort

They were randomly divided into two groups, progesterone and placebo (Figure 1), by using table of random numbers In progesterone group 250 mg of 17-α hydroxy progesterone caproate (Aburihan Co. Iran)(Femolife)injected intramuscularly (IM) every week until 36 weeks of gestation. In placebo group injections were done by a drug similar in shape and volume, but without progesterone which was prepared by the same company as placebo.Consent were taken from all patient in oral or written way. 

All injections have done by trained nurses who were blinded about the type of drugs. In the beginning each women learned the symptoms of preterm labor (PTL) and followed by telephone. The patients and statistical analyzer both were blind about the drug or placebo. A trained nurse asked them the symptoms of preterm labor (such as; labor pain; premature rupture of membrane; vaginal bleeding and backache) by phone every week from 16^th^ weeks of gestation. 

Demographic data including age, weight and height, history of smoking during recent pregnancy, duration and type of infertility, and gravidity were asked for each person. All women followed and investigated for progesterone side effects like diabetes mellitus, hypertension, and local side effects. Etiology of preterm labor (spontaneous PTL; Premature rupture of membrane (PROM); elective preterm Labor of pregnancy due to hypertension, and oligohydramnios) were asked by the trained nurse.

This study was supported by Abureihan Pharmacy Company. All steps of our work confirmed by ethical committee of Yazd Research and clinical center for Infertility.

**Statistical analysis**

All data gathered in a check list which was prepared by investigators and analyzed by SPSS software, Chi-Square, Fisher Exact test, Manner Witney , and student’s *t*- test. P<0.05 mentioned as significant difference between groups.

## Results

Among 106 pregnant women who entered in our investigation; seven women excluded from our study; two of them excluded because of cerclage, and the other omitted because of problems in follow up and continuing drug usage. So, 52 women in progesterone group and 47 ones in control group were followed and their data were analyzed (Figure 1). 

Mean±SE in progesterone group reported 30.32±4.5 years old and in placebo group was 29.06±4.94 years old (p=0.22). Duration of infertility in progesterone and placebo group reported 7.8±4.81 and 7.57±3.66 year respectively (p=0.908 Manner Witney Test). Mean age and duration of infertility were similar in both placebo and case group. In progesterone group, there was one woman with history of five pregnancies and intra uterine fetal death (IUFD), two women in gravid three, 13 women with gravid two, and 36 women with gravid one. 

In placebo group, there were 3 cases with gravid three, 2 women in gravid two, and 40 women in gravid one. So, Progesterone and placebo group were the same as gravidity (p=0.11). Cases divided into four groups according to their body mass index (BMI). In BMI less than 18 there were 4 women in progesterone group, while it was three in placebo group. BMI 18-25; there were 18 women in progesterone group and 16 women in the other group. BMI 25-30; there were 29 women in progesterone group and 21 women in placebo group. BMI more than 30; there were 5 women in progesterone group. But this differences was not statistically significant (p=0.61).

Demographic characteristic of both groups such as BMI; smoking history; gravidity; and etiology of infertility (female or male) were the same in both groups. In case group, infertility because of male problems reported in 18 women, while for female problems reported in 25 ones. These data reported in control group 18 and 19 respectively. Two groups did not have significant different according to their causes of infertility (p=0.24). Preterm labor in weeks 34-37, reported in three women in case group and 8 ones in control group. In both groups there was one woman who delivered in 32-34 weeks. Both groups did not have significant differences according to their gestational age (p=0.203) (Table I).

The risk of preterm labor in placebo group was 2.48 higher than control group that was not significant (CI: 0.81-9.94). Among causes of preterm labor, spontaneous ones were seen in 3 women in placebo group, while there was no one in progesterone group. There were 4 PROM in progesterone group and two ones in placebo group. There was no significant difference between drug and placebo group. (p=0.06) (Table II). 

There was an IUFD in placebo group, while it did not report in progesterone group. Weight of new born infants reported 3263.26±463.2 grams for test group, while it was 3053.91±466.22 grams for control group. There was a significant differences between groups according to their infants' body weight (p=0.028). In progesterone group, 11 women suffered from Gestational Diabetes Mellitus (GDM), while it was 6 women in placebo group. Gestational hypertension reported in placebo and progestrone drug group 2.1% and 15.4% respectively. Local side effects because of intra muscular injection including itching, edema and abscess was seen in placebo group (25 women) more than progesterone group (6 women). 

There was a significant difference between groups according to their side effects (p=0.01) (Table III). Low Birth Weight (LBW) reported in four women (8.7%) of placebo group, while it was two ones (3.8%) in progesterone group. and there was no significance difference between groups (p=0.41). NICU admission reported in five women (9.6%) in progesterone group, but it was nine ones (19.1%) in placebo group and there was no significance between groups (p=0.24). Cesarean reported in 51 women in test group, while it was 38 ones in control group (p=0.06) (Table IV). Mean±SD of birth weight in case group was 3263.26 (with SD=463.2) and It was more than in control group: 3053.91 (with SD=466.22. 

**Table I:** Comparison between groups according to their gestational age at birth

** Gestational age**			**More than 37 weeks**	**34-37 weeks**	**32-34 weeks**
**Group**					
Case (progesterone)					
	Number		1	3	48
	Percentage		1.9%	5.8%	92.3%
Control (placebo)					
	Number		1	8	38
	Percentage		2.1%	17%	80.9%

Chi-square Test. (p<0.05 was meaningful). (p=0.203)

**Table II:** Comparison between groups according to their causes of preterm labor

** Causes of preterm labor**			**Spontaneous preterm labor**	**PROM**	**Preterm elective termination of pregnancy**	**Term labor**
**Group**						
Case (progesterone)						
	Number		0	4	0	48
	Percentage		0%	7.7%	0%	92.3%
Control (placebo)						
	Number		3	2	3	39
	Percentage		6.4%	4.3%	6.4%	83%

Chi-square Test. (p<0.05 was meaningful) (p=0.06)

**Table III:** Comparison between groups according to their overal side effect of drug

** Side effects of drug**			**Diabetes mellitus**	**Hypertension**	**Local side effects**	**No side effects**
**Group**						
Case						
	Number		11	8	6	27
	Percentage		21.2%	15.4%	11.5%	51.9%
Control						
	Number		6	1	25	15
	Percentage		12.8%	2.1%	53.2%	31.9%

Chi-Square test. (p<0.05 was meaningful) (p=0.01)

**Table IV:** Comparison between groups according to low birth weight, NICU admission, natural vaginal delivery

** Variable**			**Low Birth Weight**	**NICU admission**	**Natural Vaginal Delivery**
**Group**					
Case (progesterone)					
	Number		2	47	1
	Percentage		3.8%	90.4%	1.9%
Control (placebo)					
	Number		4	38	9
	Percentage		8.7%	80.9%	19.1%
p-value			0.41	0.24	0.06

LBW (chi-squre test. p=0.41). NICU admission (Fisher Exact Test. p=0.24). Natural vaginal Delivery (Fisher Exact Test. p=0.06).

## Discussion

Preterm birth management and prevention remained as one of the problems in health policies and it increases neonatal mortality and morbidity rate. While the exact mechanism of both term and preterm labor remains unclear, using progesterone is a therapeutic modality which plays an important role in the progression of pregnancy because of uterine quiescence (2). This study was designed to define the effects of 17- hydroxy progesterone caproate on decreasing second trimester abortion and preterm labor in women who undergone ART. Both groups did not have significant difference in parameters like age, duration of infertility, gravidity, smoking, BMI and causes of infertility. 

These parameters were mentioned and fixed because in our study they were able to play role of confounding factors. Prevalence of preterm birth was declared in our study 13% which was more than its prevalence in normal population and it was because of infertility treatment and undiagnosed factors in women who treated by IVF. This was similar to Wisborg *et al* study (17). No mortality, because of immaturity, was seen in our study. There was just one IUFD in placebo group which was due to creasing blood pressure in mother and did not have association with progesterone or placebo usage. In our survey also there was no abortion in second trimester; it may be due to our study design which uterine anatomical malformations and cervical incompetency excluded from our study. 

It also may be related to small sample volume in our investigation. Preterm labor in gestational age more than 32 weeks were more in placebo group, but it was not significant. There was not preterm birth before gestational age of 32 weeks in both groups and it was against Wisborg *et al* and Meis *et al* results (16-18). Late preterm labor (34-37) weeks and also preterm labor (32-34) weeks of gestational age were more in placebo group but it was not significant (p=0.203). 

These evidence were against of study of Roberto *et al* and also against of Meis study. Their studies were done with larger samples than our study. Risk of preterm labor in placebo group was 2.48 more than progesterone group (CI: 0.81-9.94). It seems that, with larger samples; we have meaningful decrease in preterm labor with progesterone (16-18). In our study, spontaneous preterm labor was seen in placebo group more than progesterone group and PROM declared as the most cause of preterm birth in progesterone group. Similar to Roberta *et al* and Meis *et al* the rate of spontaneous preterm labor showed reduction after progesterone injection, which was insignificant (17, 18). In our survey, complication of placebo group was significantly higher than progesterone group (p=0.01). 

Local side effects (itching ‘edema’ and erythema) were higher in placebo groupthan the placebo group, but gestational diabetes mellitus (GDM) and hypertension were higher in progesterone group where compared to placebo group. In Meis *et al* research, local side effects in test group were more than control one. Increasing in GDM rate in progesterone group was the same as Water *et al* study, but did not confirmed by Gymfi *et al* study which association of GDM and progesterone was rejected by a clinical trial (7, 25). 

Infant’s body weight was seen in progesterone group more than placebo group that it was like Meis *et al* study, but body weight of infants in our study was more than Bahadori *et al* results (18, 21). Frequency of LBW in placebo group was more than progesterone group but it was not significant (p=0.41): and it was against of Kirston *et al* and Bahadori *et al* (21, 23). Rate of NICU admission in placebo group was more than progesterone group in our survey that it was like Mason *et al* study (24). 

There was no significant difference between both groups for cesarean which it was similar to Meis *et al *investigation. Normal Vaginal Delivery (NVD) was seen in 10 women of our study, but it was not declared by their physicians.

## Conclusion

It can be concluded, 17-hydroxy progesterone caproate was not shown significant decrease in preterm labor in ART pregnancies but it may be useful in such a high risk population according to absence of any important side effect and decreasing risk of preterm labor in comparison with placebo. This means, 17-hydroxy progesterone caproate may be able to improve the situation of ART pregnancies and decrease morbidity of preterm labor. Larger studies should be undertaken to establish usage of this drug in ART pregnancies.

## Conflict of interest

Neither my colleagues nor I were financially interested in the study.
