# Corrigendum to “Efficacy of 17 α- hydroxy progestrone on decreasing preterm labor in ART pregnancies: A randomized clinical trial” [Iran J Reprod Med 2013; 11: 785-790]

**DOI:** 10.18502/ijrm.v20i5.11176

**Published:** 2022-06-08

**Authors:** Abbas Aflatoonian, Hoora Amouzegar, Razieh Dehghani Firouzabadi

The authors have been informed of some errors that occurred in the published paper. The errors are listed as:

• A paragraph has been added to Materials and Methods section about blinding.
• The statistical test which has been used in tables I, II has been changed from chi-square test to Fischer exact test.
• The study results were re-analyzed, and some p-values were modified in tables.
• The last paragraph of discussion section has been removed.

The authors wish to apologize for these errors. The online version of the article has been updated on May 30, 2022, and can be found at http://dx.doi.org/10.18502/ijrm.v11i10.356.

